# Artificial Intelligence Discusses the Role of Artificial Intelligence in Translational Medicine: A *JACC: Basic to Translational Science* Interview With ChatGPT

**DOI:** 10.1016/j.jacbts.2023.01.001

**Published:** 2023-01-18

**Authors:** Douglas L. Mann

**Affiliations:** Department of Internal Medicine—Cardiovascular Division, Washington University School of Medicine, St. Louis, Missouri, USA

**Keywords:** artificial intelligence, chatbot, translational medicine

Like many people, I was completely taken in and simultaneously taken aback by the public release of ChatGPT (GPT stands for “generative pretrained transformer”), the natural language processing tool that allows one to have a personalized conversation with an artificial intelligence (AI) bot capable of providing detailed responses to questions (prompts) with uncanny speed. ChatGPT is built by OpenAI, a nonprofit San Francisco–based AI research and deployment company, whose mission is to “ensure that artificial general intelligence benefits all of humanity.” OpenAI also released DALLE•2, which is an AI art generator.

The development of user-friendly versatile AI chatbots has been slow in coming. As noted by Kevin Roose, a technology columnist for *The New York Times*, the responses of earlier generations of AI chatbots were often restricted to narrow well-defined tasks or questions. When the chatbot was asked to respond to questions or tasks outside of the preprogrammed AI comfort zone, the bot often “flailed.”^1^ However, in recent years, the technology has improved substantially. ChatGPT is based on GPT-3.5, which is an AI text generator that uses Reinforcement Learning from Human Feedback to inform its language model. GPT-3.5 employed human AI trainers who engaged in conversations in which they played the role of the user and the AI assistant to incorporate human responses into the machine learned responses. Although this technology is not new to AI researchers, the release of ChatGPT by OpenAI was the first time that this type of technology became freely available to the public. Making the software publicly available allowed OpenAI to register over 1 million users, who then provided human feedback on a massive scale to further refine ChatGPT’s natural language responses.

As an editor of a translational journal and as someone who has previously written about the potential perils of AI technology in health care delivery,^2^ I decided to take ChatGPT out for a test drive by prompting (ie, interviewing) the AI chatbot to engage in a point counter point discussion about the role of AI in translational medicine. To accompany this paper translational perspectives article, I also generated an Andy Warhol–inspired AI-generated picture of a female and male translational scientist (Figure 1), which was created using a commercial AI content generator (Jasper) that uses the DALL-E 2 system developed by OpenAI.

**Figure 1:**
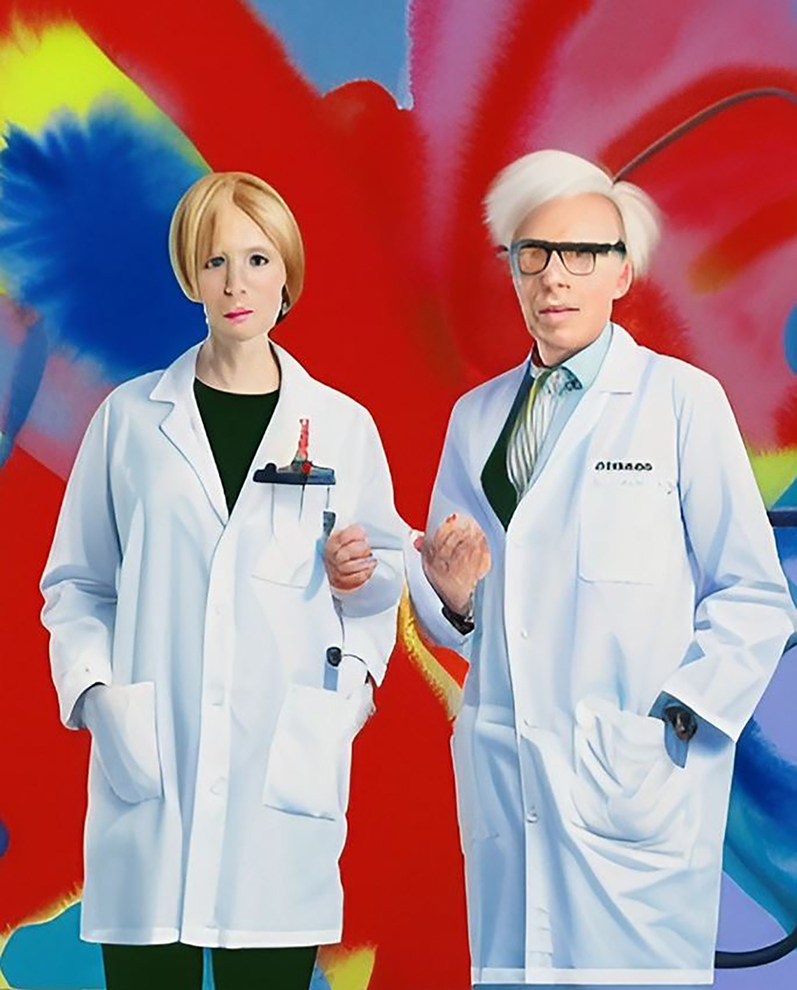
AI Generated Image of a Female and Male Translational Scientists This figure was created using a commercial AI content generator (Jasper) that uses the DALL-E 2 system developed by OpenAI.

The AI Chatbot responses that follow below were generated in response to my questions, and were copied verbatim and have not been edited for grammar, style, or content.

## Why Is Artificial Intelligence the Future of Translational Medicine?

Artificial Intelligence (AI) is rapidly becoming a key player in the field of translational medicine, and it is likely to play an increasingly important role in the future. Translational medicine is a rapidly growing field that aims to bridge the gap between basic research and clinical practice, by translating findings from the laboratory into practical applications that can improve patient care. AI is well-suited to this task, due to its ability to analyze large amounts of data quickly and accurately, and to identify patterns and trends that might not be apparent to humans.

One of the key ways in which AI is being used in translational medicine is in the analysis of large datasets, such as electronic health records or genomic data. By analyzing these datasets, AI algorithms can identify patterns and trends that might not be apparent to humans, and can help to identify potential risk factors for diseases or to predict patient outcomes. For example, AI algorithms have been used to identify risk factors for diseases such as diabetes and heart disease, and to predict patient outcomes for conditions such as sepsis and pneumonia.

Another area where AI is being used in translational medicine is in the development of personalized medicine. Personalized medicine is an approach to healthcare that is tailored to the specific needs and characteristics of individual patients, and it is becoming increasingly important as we learn more about the genetic and environmental factors that contribute to different diseases. AI algorithms can be used to analyze patient data, including genomic data, and to identify personalized treatment approaches that are likely to be most effective for individual patients.

AI is also being used in the development of new drugs and treatments. By analyzing large datasets of chemical compounds, AI algorithms can identify potential candidates for drug development, and can also help to identify potential side effects or interactions with other drugs. This can help to speed up the drug development process and reduce the cost of bringing new drugs to market.

In conclusion, AI is likely to play an increasingly important role in the future of translational medicine, due to its ability to analyze large datasets quickly and accurately, to identify patterns and trends, and to help develop personalized treatments and new drugs. As AI technologies continue to advance, it is likely that we will see even more applications of AI in the field of translational medicine, which will have the potential to improve patient care and outcomes.

## Why Will Artificial Intelligence not Be the Future of Translational Medicine?

While Artificial Intelligence (AI) has been hailed as a promising new tool in the field of translational medicine, there are a number of reasons why it is unlikely to be the future of this field. Translational medicine is a rapidly growing field that aims to bridge the gap between basic research and clinical practice, by translating findings from the laboratory into practical applications that can improve patient care.

One of the main limitations of AI in translational medicine is that it is only as good as the data that it is given. If the data that is used to train AI algorithms is biased or incomplete, the algorithms will be biased or incomplete as well. This can lead to inaccurate or misleading results, which could have serious consequences for patient care. For example, if an AI algorithm is used to predict patient outcomes and the data that it is given is biased, it may not accurately reflect the experiences of all patients, which could lead to inadequate or inappropriate treatment.

Another limitation of AI in translational medicine is that it is not capable of understanding the complexity of biological systems. While AI algorithms can analyze large amounts of data quickly and accurately, they do not have the ability to understand the underlying biological mechanisms that are at work in the body. This means that they are not able to provide a complete understanding of the factors that contribute to a particular disease or condition, and they may not be able to accurately predict patient outcomes or identify the most effective treatments.

Finally, there are also concerns about the ethical implications of using AI in translational medicine. AI algorithms are only as ethical as the people who design them, and there is a risk that they could be used to discriminate against certain groups of people, or to prioritize the interests of some patients over others. This could lead to further inequalities in healthcare, and could undermine the trust that patients have in the medical profession.

In conclusion, while AI has the potential to be a useful tool in the field of translational medicine, it is unlikely to be the future of this field. There are a number of limitations to the use of AI in this context, including the potential for bias and incomplete data, the inability to understand the complexity of biological systems, and the ethical implications of using these technologies. As a result, it is important to be cautious about relying too heavily on AI in the field of translational medicine, and to ensure that it is used in a responsible and ethical manner.

## An Editor’s Perspective on AI Chatbots

After reading the arguments for and against the role of AI in translational medicine, I found that both sets of arguments were factual and to some extent compelling. However, what each of the responses lacked was the type of nuanced insight that a person with in-depth knowledge of the field might provide. Indeed, the responses generated by the chatbot in response to my prompts were comparable to what an early career graduate student or first- or second-year medical student could generate after performing a 60- to 90-minute internet search using Google. With that said, I should acknowledge that the chatbot might have produced more nuanced responses had I known what additional prompts to provide to enhance the performance of the bot. In a 2022 Expert Survey on Progress in AI, 50% of AI experts estimated that an AI system capable of human-level thinking (referred to as Artificial General Intelligence or AI singularity) would be available by 2059.^3^ Given that this date is well beyond my tenure as Editor-in-Chief for *JACC: Basic to Translational Science*, I am reasonably confident that I will be able to hold onto my editorial position and that I will not be replaced by a Editor-in-Chief chatbot in the near term.

Although AI chatbots may not be capable of human level thinking today, I do believe that AI-generated journalistic content will become an increasingly important voice in scientific and medical journals in the not too distant future, because of the ability of the current generation of AI bots to generate reasonably curated content with blazing speed. As a case in point, ChatGPT generated the content for this paper in <1/1,000th the time that it took for me to write this article. As with any new technology, there will be limitations, which not surprisingly are covered very nicely in the countervailing argument about the limitations of AI in translational medicine. In closing, I would welcome your thoughts on the role of that AI chatbots will play in translational science, either through social media (*#JACC:BTS*) or by e-mail (jaccbts@acc.org).

## Funding Support and Author Disclosures

The author has reported that he has no relationships relevant to the contents of this paper to disclose.
